# Synthetic miR-143 Inhibits Growth of HER2-Positive Gastric Cancer Cells by Suppressing KRAS Networks Including DDX6 RNA Helicase

**DOI:** 10.3390/ijms20071697

**Published:** 2019-04-05

**Authors:** Yoshihisa Tokumaru, Toshihiro Tajirika, Nobuhiko Sugito, Yuki Kuranaga, Haruka Shinohara, Takuya Tsujino, Nobuhisa Matsuhashi, Manabu Futamura, Yukihiro Akao, Kazuhiro Yoshida

**Affiliations:** 1 United Graduate School of Drug Discovery and Medical Information Sciences, Gifu University, 1-1 Yanagido, Gifu 501-1194, Japan; yoshitoku1090@gmail.com (Y.T.); v3501002@edu.gifu-u.ac.jp (N.S.); v3501001@edu.gifu-u.ac.jp (Y.K.); harukashinohara313@gmail.com (H.S.); uro061@osaka-med.ac.jp (T.T.); 2 Department of Surgical Oncology, Graduate School of Medicine, Gifu University, 1-1 Yanagido, Gifu 501-1194, Japan; t-tazi@live.jp (T.T.); nobuhisa@gifu-u.ac.jp (N.M.); mfutamur@gifu-u.ac.jp (M.F.); kyoshida@gifu-u.ac.jp (K.Y.); 3 Department of Urology, Osaka Medical College, 2-7 Daigaku-machi, Takatsuki, Osaka 569-8686, Japan

**Keywords:** miR-143, HER2-positive gastric cancer, KRAS, DDX6

## Abstract

Gastric cancer (GC) is one of the most common cancers worldwide. In the clinical setting, the identification of HER2 overexpression in GC was a significant finding, as trastuzumab, an anti-HER2 drug, provides a survival advantage to HER2-positive GC patients. In HER2-postive GC, the dysregulation of PI3K/AKT and MAPK/ERK signaling pathways has been reported, and inhibition of these pathways is an important therapeutic strategy. MiR-143 is known to act as a tumor suppressor in several cancers, such as bladder cancer, breast cancer, colorectal cancer, and gastric cancer. In the current study, we developed a novel chemically-modified miR-143 and explored the functions of this synthetic miR-143 (syn-miR-143) in HER2-positive gastric cancer. The expression level of miR-143 was down-regulated in GC cell lines, including HER2-positive GC cell lines, MKN7, and KATO-III. The ectopic expression of miR-143 in those cell lines suppressed cell growth through systemic silencing of KRAS and its effector signaling molecules, AKT and ERK. Furthermore, syn-miR-143 indirectly down-regulated the expression of HER2, an upstream molecule of KRAS, through silencing DEAD/H-box RNA helicase 6 (DDX6), RNA helicase, which enhanced HER2 protein expression at the translational step in HER2-positive GC cells. These findings suggested that syn-miR-143 acted as a tumor suppressor through the impairment of KRAS networks including the DDX6.

## 1. Introduction

Gastric cancer (GC) is one of the most common causes of cancer-related deaths worldwide, being the third in men and fifth in women. In addition, approximately 720,000 deaths are related to GC annually [1]. The incidence and mortality have declined in many countries worldwide due to improvements in diagnosis and staging techniques; however, the survival rate still remains poor for this disease [2,3]. 

Human epidermal growth factor receptor 2 (HER2) is associated with the poor outcome of various cancers, such as advanced gastric cancer and gastroesophageal junction cancer [4]. The overexpression of HER2 in GC is observed in approximately 20% of cases [5]. Trastuzumab is a molecular-targeting drug for HER2; and as the result of the ToGA trial, the addition of trastuzumab to chemotherapy has become the standard treatment for advanced HER2-positive GC patients and is the only anti-HER2 agent approved for treatment of such patients nowadays [6,7]. Regarding the growth-related signaling pathways operating in HER2-positive gastric cancer, the dysregulation of PI3K/AKT signaling pathways is frequently involved in the mechanism of resistance to trastuzumab [8]; and increased activity of MAPK/ERK signaling pathways results in invasion and metastasis in GC [9]. Therefore, the control of both of these pathways is very critical as a strategy for developing novel agents to treat HER2-positive GC cells. 

MicroRNAs (miRNAs) are endogenous small RNAs, 17–25 nucleotides in length, that repress protein expression by binding to the 3′ untranslated region (3′UTR) in the mRNA of their target gene at the translational level [10]. MiR-143 functions as an anti-oncomir in several cancers, such as bladder cancer [11], breast cancer [12], colorectal cancer [13], and gastric cancer [14].

Previously, we reported the anti-tumor effect of a novel synthetic miR-143 (syn-miR-143) on colorectal cancer, which miR acts on by suppressing the KRAS signaling networks in colorectal cancer cells [15]. In addition, we recently found the regulatory function of DEAD-box RNA helicase 6 (DDX6) in the expression of HER2 at the post-transcriptional level in GC cells [16]. In the current study, we clarified the inhibitory effect of syn-miR-143 on the KRAS signaling networks in HER2-positive GC by targeting KRAS and its effector molecules, AKT and ERK. Furthermore, we found that syn-miR-143 indirectly regulated HER2 expression by silencing DDX6 in HER2-positive GC cells. Our data indicated a novel therapeutic strategy against HER2-positive gastric cancer involving perturbation of KRAS networks including the DDX6 mediated by tumor suppressor miR-143.

## 2. Results

### 2.1. Expression Levels of miR-143 Were Down-Regulated in Gastric Cancer Cell Lines

**Figure 1:**
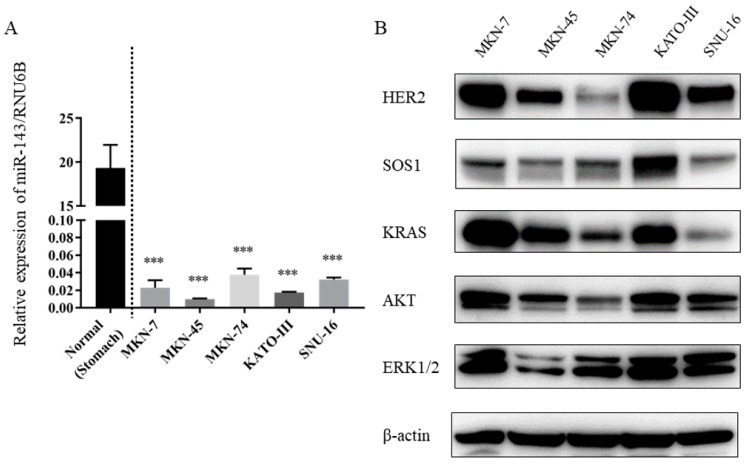
Down-regulation of miR-143 in gastric cancer cell lines, and up-regulation of HER2, KRAS, and KRAS-related molecules in HER2-positive gastric cancer cell lines MKN-7 and KATO-III. (**A**) Relative expression level of miR-143 in gastric cancer cell lines. (**B**) HER2-positive gastric cancer cell lines MKN-7 and KATO-III showed up-regulated KRAS and its effector signaling proteins ATK and ERK compared with their levels in other gastric cancer cell lines. In addition, the protein expression levels of key molecules of the KRAS networks were up-regulated in MKN-7 and KATO-III cells. Results are presented with the mean ± SD; *** *p* < 0.001.

Firstly, in order to analyze the expression levels of miR-143 in the gastric cancer cells used in this study, we performed qRT-PCR using TaqMan primers and probes. The results showed that the expression level of miR-143 was significantly down-regulated in all of the gastric cancer cell lines tested, compared with its level in the normal epithelial tissue (Figure 1A).

### 2.2. Expression Levels of KRAS and Downstream Molecules Were Up-Regulated in HER2-Positive Gastric Cancer Cell Lines

We investigated the expression level of HER2 in the gastric cell lines by performing Western blotting (WB). As shown in Figure 1B, the expression of HER2 was extremely high in MKN-7 cells, which display HER2 gene amplification, and in KATO-III cells, in which FGFR2 gene amplification occurs, when compared with the expression in MKN-74 cells, having no gene amplification of receptor of tyrosine kinases including HER2. In addition, the expression levels of downstream molecules such as KRAS, AKT, and ERK were up-regulated in MKN-7 and KATO-III cells compared with those of the other gastric cancer cell lines examined (Figure 1B). Regarding KRAS mutation, both MKN-7 and KATO-III cells do not harbor any mutation of KRAS.

Compared with that in HER2-positive breast cancer cell line SKBR-3, the expression levels of HER2 in HER2-positive gastric cancer cell lines MKN-7 and KATO-III were considerably lower. (Supplementary Figure S1). However, the expression level of KRAS in HER-2 gastric cancer cell lines was higher than that in the SKBR3 cell line (Supplementary Figure S1).

The inverse correlation between miR-143 and HER2 or KRAS was not significant, but there was a tendency for such a correlation (Supplementary Figure S1).

Since we elucidated the relationship between HER2 overexpression and the downstream transduction via miR-143, we focused on MKN-7 and KATO-III cells for further study.

### 2.3. Ectopic Expression of miR-143 Inhibited the Growth of MKN-7 and KATO-III Cells by Targeting KRAS and Its Related Signaling Molecules

**Figure 2:**
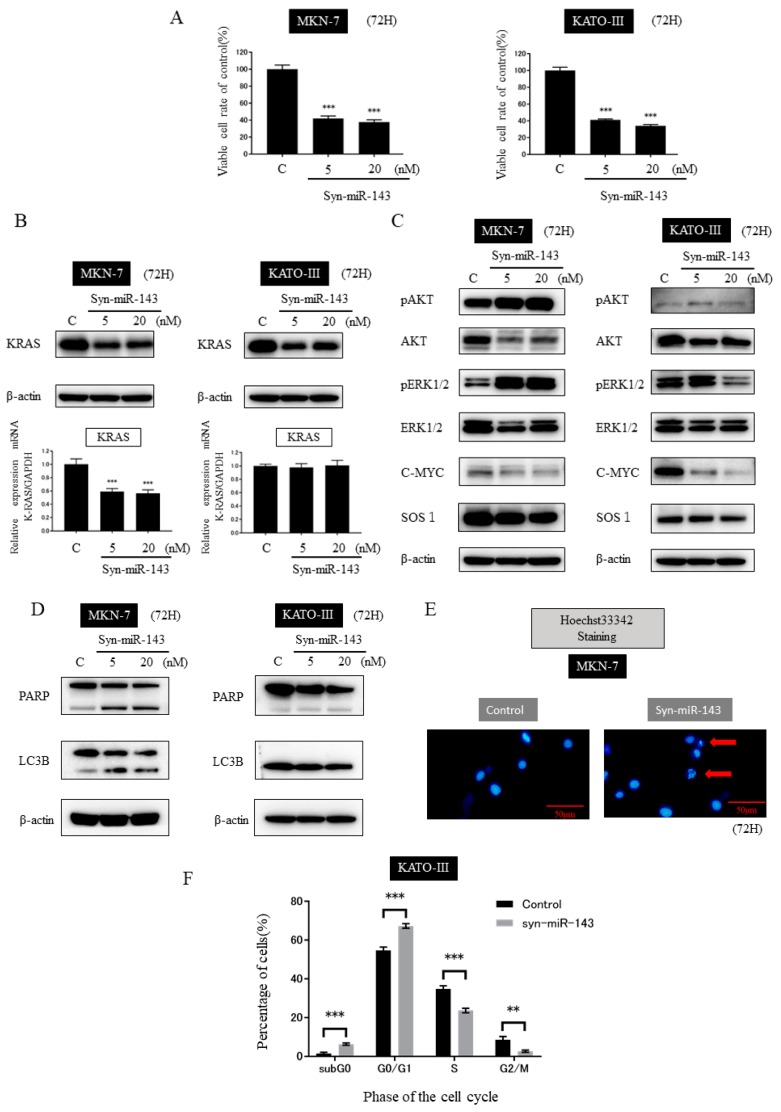
Ectopic expression of miR-143 in gastric cancer cells MKN-7 and KATO-III. (**A**) Cell viability at 72 h after transfection of MKN-7 and KATO-III cells with control RNA or synthetic miR-143 syn-miR-143. (**B**) Western blot analysis and qRT-PCR of KRAS expression at 72 h after transfection with control RNA (20 nM) or syn-miR-143 (5 nM, 20 nM). Densitometric values of KRAS/β-actin were calculated, and the values of controls are indicated as “1”. (**C**) Western blot analysis of the expression levels of AKT, pAKT, ERK1/2, pERK1/2, C-MYC, and SOS1 at 72 h after transfection with control RNA (20 nM) or syn-miR-143 (5 nM, 20 nM). (**D**) Western blot analysis of PARP and LC3B at 72 h after transfection with control RNA (20 nM) or syn-miR-143 (5 nM, 20 nM). (**E**) Hoechst 33342 staining of MKN-7 cells at 72 h after transfection with control RNA (20 nM; left) or syn-miR-143 (20 nM; right). The typical apoptotic features, such as chromatin condensation and fragmentation of nuclei, were observed (red arrows). (**F**) Cell-cycle analysis of KATO-III cells after transfection with control RNA (5 nM) or syn-miR-143 (5 nM). Results are presented with the mean ± SD; ** *p* < 0.01; *** *p* < 0.001.

To investigate the effect of miR-143 on HER2-positive gastric cancer cells, we transfected MKN-7 and KATO-III cells with syn-miR-143. The ectopic expression of miR-143 in both cell lines significantly reduced the number of viable cells (Figure 2A). These results suggested that miR-143 functioned as a tumor suppressor microRNA (TS-miR) in HER2-positive gastric cancer. We considered that this inhibition of cell growth was due to suppression of KRAS networks by miR-143. Therefore, we next examined the expression levels of KRAS by performing WB and qRT-PCR. The expression level of KRAS protein in both cell lines was down-regulated by the transfection with syn-miR-143 (Figure 2B). In addition, in MKN-7 cells the down-regulation of KRAS was observed even at the mRNA level, which did not occur in the KATO-III cells (Figure 2B). Subsequently, we examined the expression levels of the effector molecules of KRAS by performing WB. The down-regulation of AKT, ERK, and c-MYC proteins was observed in MKN-7 and KATO-III cells (Figure 2C). The expression levels of pAKT and pERK were up-regulated in MKN-7, but not in KATO-III, cells (Figure 2C). Regarding SOS1, the expression level of its protein was also decreased in MKN-7 and KATO-III cells. Thus, these findings were similar to those made in the case of colon cancer cells [15].

### 2.4. Transfection of syn-miR-143 Induced Apoptosis in MKN-7 Cells and Cell-Cycle Arrest in KATO-III Cells

Regarding cell death, the introduction of syn-miR-143 up-regulated the expression levels of cleaved PARP and the second form of LC3B in MKN-7 cells (Figure 2D). In KATO-III cells, however, this transfection had no effect on the expression of those molecules (Figure 2D). Furthermore, we performed Hoechst33342 staining to observe apoptosis in MKN-7 cells. In MKN-7 cells, the fragmentation of nucleus was observed in cells treated with syn-miR-143 (Figure 2E). To clarify the reason why cell growth suppression was induced in KATO-III cells when they had been transfected with syn-miR-143, we performed image cytometry. The results of cell-cycle analysis revealed cell-cycle arrest at the G1 phase (Figure 2F).

### 2.5. KRAS and Its Related Molecules are Targets of syn-miR-143

**Figure 3:**
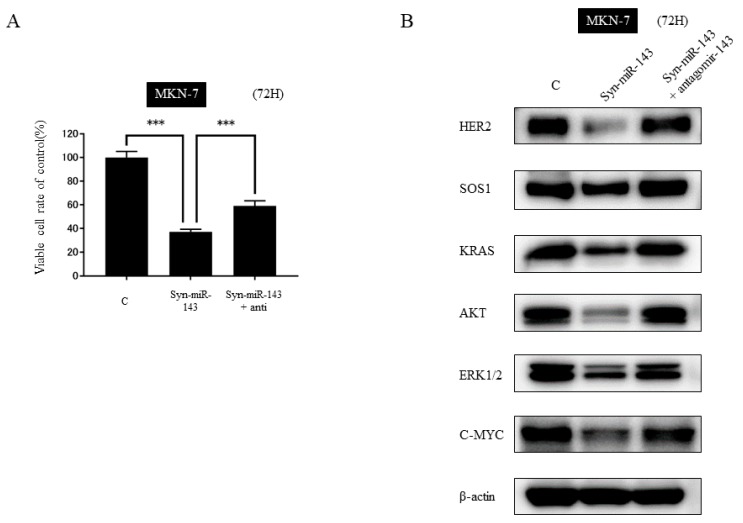
Antagomir assay of miR-143 in MKN-7 cells. (**A**) Cell viability at 72 h after transfection with control RNA alone (5 nM), syn-miR-143 alone (5 nM), or the combination of syn-miR-143 (5 nM) and antagomir-143 (10 nM). (**B**) Western blot analysis of SOS1, KRAS, AKT, ERK1/2, and C-MYC at 72 h after transfection with control alone, syn-miR-143 alone, or the combination of syn-miR-143 and antagomir-143. Results are presented with the mean ± SD; ***, *p* < 0.001.

To verify that KRAS and its related molecules were targets of syn-miR-143, we performed an antagomir assay. MKN-7 cells were co-transfected with syn-miR-143 and anti-miR-143. The significant cell growth suppression due to the transfection with syn-miR-143 was not fully restored but was recovered by co-transfection with antagomir-143 (Figure 3A). The protein expression was fully restored by co-transfection with antagomir-143 (Figure 3B).

These findings taken together confirmed that syn-miR-143 could perturb KRAS networks systematically.

### 2.6. DDX6 Is a Direct Target Gene of syn-miR-143 and syn-miR-143 Down-Regulated the Expression Level of HER2 through Targeting DDX6

**Figure 4:**
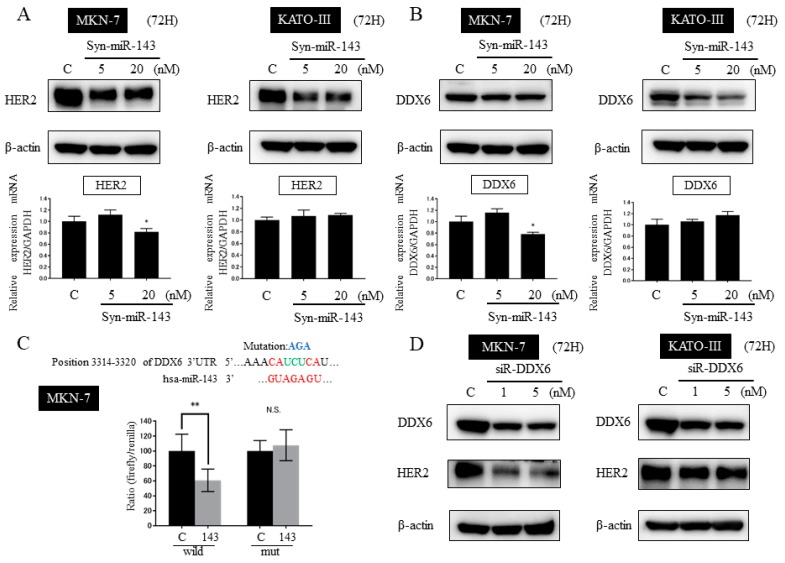
Relationship between HER2 and DEAD/H-box RNA helicase 6 (DDX6) expression after transfection with syn-miR-143. (**A**) Western blot analysis and qRT-PCR of HER2 at 72 h after transfection of MKN-7 and KATO-III cells with control RNA (20 nM) or syn-miR-143 (5 nM, 20 nM). (**B**) Western blot analysis and qRT-PCR of DDX6 at 72 h after transfection of MKN-7 or KATO-III cells with control RNA (20 nM) or syn-miR-143 (5 nM, 20 nM). (**C**) Relative luciferase activities of wild-type and mutant binding site on DDX for miR-143 in MKN-7 cells. (**D**) Expression levels of DDX6 and HER2 at 72 h after transfection of MKN-7 or KATO-III cells with control RNA (5 nM) or siR-DDX6 (1 nM, 5 nM). Results are presented with the mean ± SD; * *p* < 0.05, ** *p* < 0.01; N.S., not statistically significant.

Next, we examined the effect of syn-miR-143 on the expression levels of HER2 protein and mRNA in MKN-7 and KATO-III cells. After the transfection with syn-miR-143, HER2 protein was significantly down-regulated in both cell lines (Figure 4A). However, at the mRNA level, no obvious down-regulation was observed for either cell line (Figure 4A). Since HER2 was not a direct target of miR-143, we sought to elucidate the mechanism underlying the down-regulation of HER2. As we had previously reported that DDX6 post-transcriptionally regulates HER2 expression [16], we next examined the expression level of DDX6. As expected, the expression level of DDX6 was significantly decreased in MKN-7 and KATO-III cells treated with syn-miR-143 (Figure 4B). In order to verify that DDX6 was a direct target of miR-143, we first used a computational search tool (Target Scan Human) to predict the binding site. Then, to confirm that DDX6 was indeed a direct target of miR-143, we performed a luciferase reporter assay. MKN-7 cells were co-transfected with luciferase reporters and either control RNA(5nM) or syn-miR-143(5nM). The relative luciferase activity of the wild type was suppressed by about 40% by the miR, and this suppression was not seen with the mutant type (Figure 4C).

To elucidate the relationship between the down-regulation of HER2 and DDX6 after the ectopic expression of miR-143, we transfected MKN-7 and KATO-III cells with siR-DDX6. The knockdown of DDX6 induced a remarkable decrease in the expression level of HER2 in both MKN-7 and KATO-III cells (Figure 4D). Moreover, the enforced overexpression of DDX6 in both cell lines promoted an increase in the expression of HER2 at the protein level but not at the mRNA level (Supplementary Figure S2A,B).

Based on these results, we concluded that syn-miR-143 directly targeted DDX6 and thus indirectly affected the expression level of HER2 through regulation of DDX6 expression.

### 2.7. Knockdown of KRAS Alone Was Not Sufficient to Suppress the Cell Growth and the Effector Signals of KRAS in HER2-Positive Gastric Cancer Cell Lines

**Figure 5:**
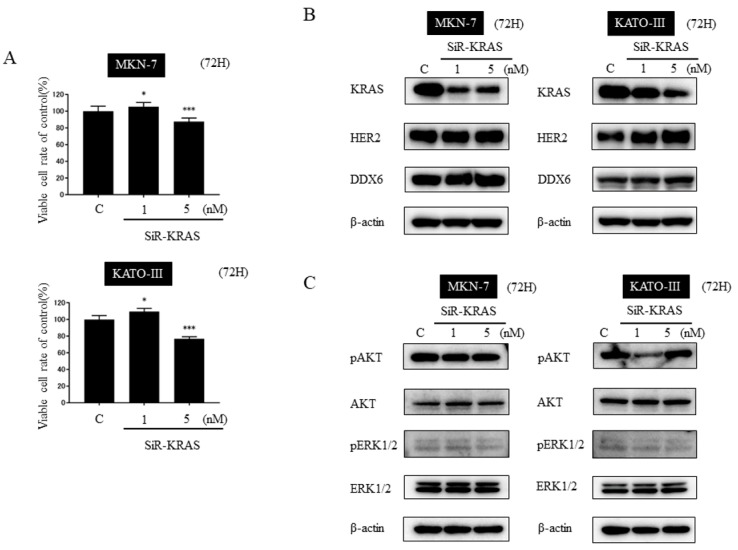
Effects of knockdown of KRAS on cell proliferation and protein expression profiles of KRAS network-related molecules. (**A**) Cell viability at 72 h after transfection of MKN-7 and KATO-III cells with control RNA (5 nM) or siR-KRAS (1 nM, 5 nM). (**B**) Expression levels of KRAS, DDX6, and HER2 at 72 h after transfection of MKN-7 and KATO-III cells with control RNA (5 nM) or siR-DDX6 (1 nM, 5 nM). (**C**) Expression levels of KRAS effector signaling molecules at 72 h after transfection of MKN-7 and KATO-III cells with control RNA (5 nM) or siR-DDX6 (1 nM, 5 nM). Results are presented with the mean ± SD; * *p* < 0.05, *** *p* < 0.001.

In the HER2/KRAS cascade, the expression level of KRAS was up-regulated in HER2-positive gastric cancer cell lines MKN-7 and KATO-III compared with that in the other cell lines (Figure 1B). To examine the effect of silencing KRAS in HER2-positive gastric cancer cell lines, we performed a cell viability assay and Western blotting after the transfection of MKN-7 and KATO-III cells with siR-KRAS. As a result, the number of viable cells was decreased approximately 20% in both cell lines (Figure 5A). The expression level of KRAS was down-regulated significantly in MKN-7 and KATO-III cells (Figure 5B). However, the down-regulation of HER2 and DDX6 was not observed in either cell line, thus differing from the results found for the transfection with syn-miR-143. In addition, the expression levels of AKT and ERK did not decrease in either cell line (Figure 5C). However, the expression levels of pAKT and pERK were down-regulated in KATO-cells, but not in MKN-7 ones (Figure 5C).

**Figure 6:**
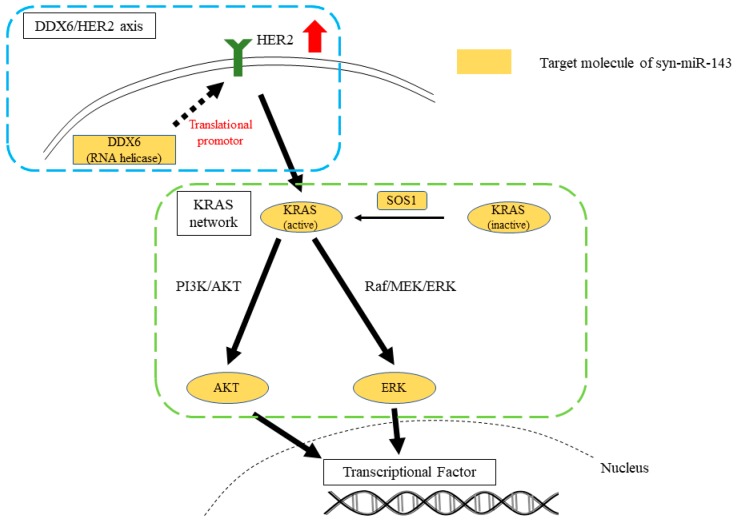
Schematic diagram demonstrating the effect of syn-miR-143 on the signaling pathways of HER2/KRAS network and HER2/DDX6 axis in gastric cancer cells. The genes colored in orange are direct targets of syn-miR-143. The dotted line and red arrow demonstrates that DDX6 enhances the HER2 protein expression.

These results indicated that knockdown of KRAS alone was not sufficient to inhibit the signaling pathways of HER2 and KRAS networks. To the contrary, syn-miR-143 effectively suppressed the cell growth by inhibiting the networks of KRAS, including the DDX6 (Figure 6).

### 2.8. Syn-miR-143 Exhibited an Anti-Tumor Effect on Xenografted Tumors in Nude Mice

**Figure 7:**
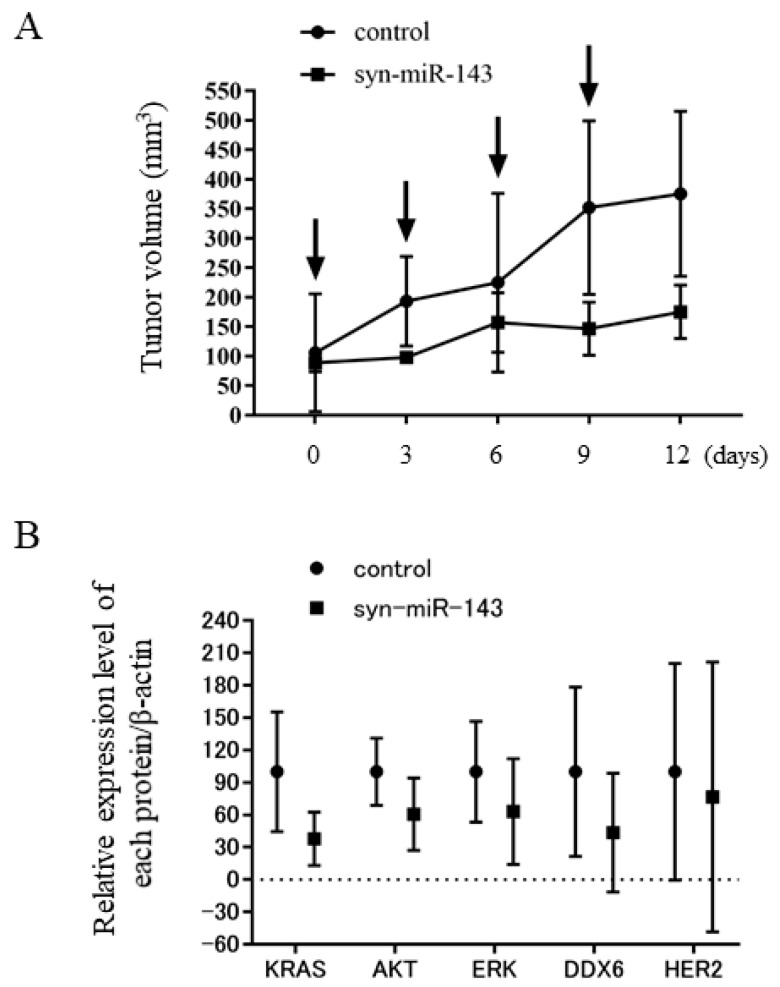
Anti-tumor effect of syn-miR-143 in vivo. (**A**) Time course of tumor size in KATO-III cell-xenografted nude mice treated with control RNA or syn-miR-143. Arrow represents an intravenous injection of control RNA (1.5 mg/kg/administration) or syn-miR-143 (1.5 mg/kg/administration) given every 3 days. (**B**) Western blot analysis of HER2, KRAS, AKT, and ERK1/2 in the samples from tumor tissues. Relative expression levels of each protein/β-actin were calculated based on densitometric values of each protein and β-actin.

To investigate the anti-tumor effect of syn-miR-143 in vivo, we inoculated KATO-III cells subcutaneously into nude mice. After confirming tumor engraftment, we systemically injected intravenously a solution containing control miR or syn-miR-143. As shown in Figure 7A, significant suppression of tumor growth was observed in mice treated with syn-miR-143. In addition, the Western blotting results revealed a tendency for a reduction in the expression levels of HER2, KRAS, and its effector molecules, AKT and ERK, even in this in vivo experiment (Figure 7B, Supplementary Figure S3A,B).

## 3. Discussion

With the remarkable advances in research on microRNAs over the past few decades, their role in carcinogenesis has been clarified in several human cancers, including gastric cancer. MicroRNA functions as an oncomir or a tumor suppressor by silencing anti-oncogene or oncogene, respectively. Several microRNAs are reported to be candidates for tumor suppressors in gastric cancer, such as let-7 and miR-143 [17,18].

In the current study, we explored the anti-tumor effect of a novel syn-miR-143 on HER2-positive GC. Our data elucidated that the suppressive effect of syn-miR-143 on HER2-positive GC is due to suppressing KRAS signaling networks downstream of overexpressed HER2.

Firstly, we demonstrated that the down-regulation of miR-143 was observed in GC cell lines, including HER2-positive GC cell lines, MKN-7 and KATO-III (Figure 1A). In addition, we investigated the effect of syn-miR-143 on HER2-negative gastric cancer cells, MKN-45 and MKN-74. Cell growth suppression was observed when transfected with syn-miR-143 into those cells (Supplementary Figure S4). From these results, we consider that miR-143 also functions as a tumor suppressor microRNA even in HER2-negative gastric cancer cells. Regarding the clinical samples, we previously reported the down-regulation of miR-143 in clinical samples taken from gastric cancer patients [19]. These findings indicated that miR-143 functions as a tumor suppressor in GC cells.

HER2 overexpression leads to the dysregulation of PI3K/AKT and/or MAPK/ERK signaling pathways, which is very critical for the proliferation and survival of cancer cells [5,20,21]. The up-regulated expression of KRAS, AKT, and ERK, which are the downstream molecules of HER2, was observed in HER2-positive GC cell lines MKN-7 and KATO-III, compared with their expression in other GC cell lines tested (Figure 1B). However, even though the elevated KRAS expression was observed in MKN-7 and KATO-III cells, the knockdown of KRAS alone resulted in insufficient cell growth suppression in these 2 cell lines because of the alternative activation of PI3K/AKT and/or MAPK/ERK signaling pathways. These results implied the critical role of KRAS networks in HER2-positive GC cells and the importance of silencing the pleural key molecules of the KRAS networks as a therapeutic strategy. A recent report of ours clarified the efficient anti-tumor effect of syn-miR-143 on colorectal cancer cells, which occurred by impairing the KRAS networks systematically [15]; and similar effects of syn-miR-143 were presently shown even in HER2-positive GC cells (Figure 2B,C).

DDX6 (also termed RCK/p54) is an oncogenic RNA helicase belonging to the family of human DEAD/H-box RNA helicases. DDX6 binds to the 5′UTR of its target mRNA and recruits ribosomes, resulting in the initiation of translation (Supplementary Figure S5). In our previous studies, we detected the overexpression of DDX6 in most of the tumor samples and cell lines examined, and found that DDX6 positively regulates c-Myc expression at the translational step in various cancers [22,23,24]. Furthermore, DDX6 also positively regulates HER2 expression at the translation step in GC cells [16]. In the present study, we demonstrated DDX6 to be a direct target of syn-miR-143 and reconfirmed the regulatory function of DDX6 in HER2 expression (Figure 4A–D, Supplementary Figure S2).

Despite the development of molecular-targeting drugs for various cancers, trastuzumab is the only approved one for HER2-positive GC. Lapatinib, an inhibitor of EGFR and HER2 receptors, could not improve overall survival (OS) in phase III studies [25,26]. This result enables us to know the problem of treatment strategy against trastuzumab-resistant HER2-positive GC. The alternative activation of the downstream signal pathways of receptor tyrosine kinases occurs in most trastuzumab-resistant HER2-positive GC [6]. Based on such a situation, we examined the effect of combined treatment with syn-miR-143 and lapatinib on HER2-positive GC cell lines MKN-7 and KATO-III. However, this combined treatment did not exhibit a synergistic effect on HER2-positive GC cells (Supplementary Figure S6). Since syn-miR-143 considerably down-regulated HER2 expression, the benefit of combination with lapatinib could not be observed. Another reason is that the syn-miR-143 had a potent anti-cancer effect on the cell viability, which led to the difficulty of demonstrating the benefit of combination treatment with lapatinib.

In conclusion, syn-miR-143 exhibited its anti-tumor effect on HER2-positive GC by impairing the KRAS networks systematically, including DDX6 RNA helicase.

## 4. Material and Methods

### 4.1. Cell Culture and Cell Viability

MKN7, MKN45, MKN74, KATO-III, and SNU-16 cells were purchased from the JCRB (Japanese Collection of Research Bioresources) Cell Bank. They were cultured in RPMI-1640 medium supplemented with 10% (*v*/*v*) heat-inactivated fetal bovine serum (FBS, Sigma-Aldrich Co., St. Louis, MO, USA) and 2 mm L-glutamine under an atmosphere of 95% air and 5% CO2 at 37 °C. The number of viable cells was determined by performing the trypan-blue dye exclusion test.

### 4.2. Cell Transfection Experiment with miRNA or siRNA

MKN-7 and KATOIII cells were seeded into 6-well plates at a concentration of 0.5 × 10^5^/well (10–30% confluence) on the day before the transfection. Syn-miR-143, siR-KRAS, and siR-DDX were used for cell transfection and performed with Lipofectamine RNAiMAX Reagent (Invitrogen, Carlsbad, CA, USA) by following the manufacturer’s lipofection protocol. The sequence of the syn-miR-143 used in this study was published previously [15]. The sequences of siRNAs for KRAS and DDX6 were the following: 5′-AAU GCA UGA CAA CAC UGG AUG ACCG-3′ (siR-KRAS) and 5′-GGA UAU UAU UCU CAC GCU ACC UAAA-3′ (siR-DDX6). As a control, we used the nonspecific miRNA (HSS, Hokkaido, Japan) sequence of 5′-GUA GGA GUA GUG AAA GGCC-3′. The effect of transfection was observed at 72 h after the introduction of the miRNA or siRNA.

### 4.3. Western Blotting Analysis

Transfected cells were collected for Western blotting analysis 72 h after transfection with miRNA or siRNA. All cells and tumor samples from xenografted nude mice were lysed for 20 min on ice with lysis buffer containing 1% Protease Inhibitor Cocktail (Sigma-Aldrich). The protein lysis buffer consisted of 10 mM Tris–HCl (pH 7.4), 1% NP4O, 0.1% deoxycholic acid, 0.1% SDS, 150 mM NaCl, and 1 mM EDTA. Polyacrylamide gels of 7.5%, 10%, and 12.5% (Wako) were used to perform SDS PAGE, and the separated proteins were then transferred to PVDF membranes (Perkin Elmer Life Sciences, Boston, MA, USA). The membranes were thereafter blocked with 5% non-fat dry milk (Cell Signaling Technology, Danvers, MA, USA) and incubated with the desired primary antibody at 4 °C overnight.

The following antibodies were used in this study: Anti-DDX6 (RCK) antibody, purchased from Santa Cruz Biotechnology (Dallas, TX, USA); anti-KRAS antibody, purchased from LifeSpan BioScience (Seattle, WA, USA); anti-HER2, anti-Sos1, anti-phospho-ERK1/2, anti-ERK1/2, anti-phospho-AKT (Ser473), anti-AKT, anti-PARP, anti-LC3B, and anti-c-Myc antibodies, obtained from Cell Signaling Technology (Santa Cruz, CA, USA); and anti-ß-actin antibody, purchased from Sigma-Aldrich (Darmstadt, Germany).

The membranes were then washed three times with PBS containing 0.1% Tween 20, incubated further with HRP-conjugated sheep anti-mouse or donkey anti-rabbit IgG antibody (Cell Signaling Technology) for 1 h at room temperature, and then washed three times with PBS containing 0.1% Tween 20. An Enhanced Chemiluminescence Detection Kit (PerkinElmer, Waltham, MA, USA) was used to visualize the immunoblots.

### 4.4. Quantitative RT-PCR

NucleoSpin (TaKaRa, Otsu, Japan) was used after DNase I treatment to isolate total RNA from cultured cells. Prime Script H RT reagent Kit (TaKaRa) was used to reverse-transcribe total RNA, in order to determine the expression levels of KRAS, DDX6, HER2, and GAPDH mRNAs. According to manufacturer’s protocols, quantitative RT-PCR was performed with Thermal cycler Dice Real Time System II (TaKaRa). PCR amplifications were triplicated by using THUNDERBIRD probe qPCR Mix (Toyobo, Osaka, Japan), following the manufacturer’s procedures. For relative quantification, the ΔΔCt method was performed. Normalization of the acquired data was performed using endogenous control U6 (RNU6B) and GAPDH. The mRNAs of KRAS, DDX6, and HER2 were detected by performing RT-PCR using primer sets for those genes. Total RNA of human stomach was obtained from Clontech Laboratories, Inc. (Mountain View, CA, USA).

The sequences of these primers were as follow: 5′-CCT GCT CCA TGC AGA CTG TTA-3′ (KRAS forward), 5′-TGG GAG GTG CCA GACT-3′ (KRAS reverse); 5′-GAC ACA GCA ACA GAT GAA CC-3′ (DDX6 forward), 5′-TGT CCT TCT TCA GGT CTA GC-3′ (DDX6 reverse); 5′-CTG ATG GGT TAA TGA GCA AAC TGA-3′ (HER2 forward), 5′-CCA AAT TCT GTG CTG GAG GTA GAG-3′ (HER2 reverse).

### 4.5. Dual luciferase Reporter Assay

We constructed a pMIR vector by inserting the sequence of a possible binding site in the 3′UTR of human DDX6 (No.3314-3320) into a pMIR-REPORT™ Luciferase miRNA Expression Reporter Vector (Applied Biosystems) according to the manufacturer’s protocol. Moreover, by using a PrimeSTAR^®^ Mutagenesis Basal Kit (TaKaRa), we also made another pMIR construct having a mutated seed sequence for miR-143 (wild type, CAUCUCA; mutant, CAAGACA). The sequence of this mutation vector was submitted to the Life Science Research Center, Gifu University for DNA sequencing. MKN-7 cells were seeded into 96-well plates at a concentration of 0.1 × 10^4^ per well on the day before the transfection. The cells were co-transfected with either reporter vector (0.01 μg/well each) and 20 nM syn-miR-143 or nonspecific non-coding siRNA (Dharmacon, Tokyo, Japan). Luciferase activities were determined by using the Dual-Glo Luciferase Assay System (Promega) and a GLOMAX 20/20 LUMINOMETER (Promega) at 24 h after co-transfection. The firefly luciferase activity was normalized by the co-transfected Renilla luciferase activity for determining co-transfection efficiency.

### 4.6. Hoechst 33352 Staining

The collection of transfected MKN-7 cell was performed at 72 h after the transfection. Hoechst 3342 (5 μg/mL) was used for the staining of the cells. The cells were cultured at 37 °C for 1 h and washed with phosphate-buffered saline. After the wash, cells were resuspended and examined with an Olympus microscope (Tokyo, Japan) equipped with an epi-illuminator and appropriate filters. The apoptotic cells were characterized by the condensed and/or fragmented nuclei.

### 4.7. Cell-Cycle Analysis

At 72 h after the transfection with syn-miR-143(5 nM) or nonspecific miRNA (5 nM), MKN-7 cells were collected and stained with propidium iodide. The stained cells were analyzed with a Tali^®^ Image-Based Cytometer according to the manufacturer’s protocol (Life Technologies, Carlsbad, CA, USA).

### 4.8. Human Tumor Xenograft Model

Animal experimental protocols were approved by the Committee for Animal Research and Welfare of Gifu University (H30-42). BALB/cSLC-nu/nu (nude) mice were obtained from Japan SLC (Hamamatsu, Japan). KATO-III cells were injected at 4 × 10^6^ cells/100 μL per site into the back of each mouse. At 7 days after the inoculation, we confirmed the engraftment of the tumor. For administration to the mice, miRNA was mixed with poly (ethylene glycol)-poly (Ornithine) copolymer (a gift from Professor Kataoka) to obtain miRNA-loaded polyionic copolymers (PIC) to deliver the miRNA to the tumors. We systemically injected intravenously a solution containing control-miR or syn-miR-143. The tumor volume was calculated by using the following formula: 0.5236 L1 (L2)^2^ (L1: the long axis, L2: the short axis of the tumor). Evaluation of the tumors was performed after killing the mice at 19 days following transplantation of the tumor cells.

### 4.9. Statistics

Each experiment was conducted in triplicate. The two-sided Student’s test was used to evaluate the statistical significances of differences. Pearson’s correlation coefficient analysis was performed in some parts of this study. Statistical significance was defined as a *p* value < 0.05. The obtained data were presented as the mean ± standard deviation.

## 5. Conclusions

Syn-miR-143 exhibited its growth inhibitory effect by silencing not only the KRAS signaling networks, but also by causing down-regulation of HER2 expression through targeting of DDX6 in HER2-positive GC both in vitro and in vivo. Our data have afforded the conclusion that a novel therapeutic strategy against HER2-positive gastric cancer would be the perturbation of KRAS networks by tumor suppressor miR-143.

## Supplementary Materials

Supplementary materials can be found at https://www.mdpi.com/1422-0067/20/7/1697/s1.

## Abbreviations

DDX6	DEAD/H-box RNA helicase 6
Syn-miR-143	synthetic miR-143
