# Supplementary figures and images for: Synthetic miR-143 Inhibits Growth of HER2-Positive Gastric Cancer Cells by Suppressing KRAS Networks Including DDX6 RNA Helicase
Source: Int J Mol Sci. 2019 Apr 5;20(7):1697. doi: 10.3390/ijms20071697 (PMC6479539; doi:10.3390/ijms20071697)

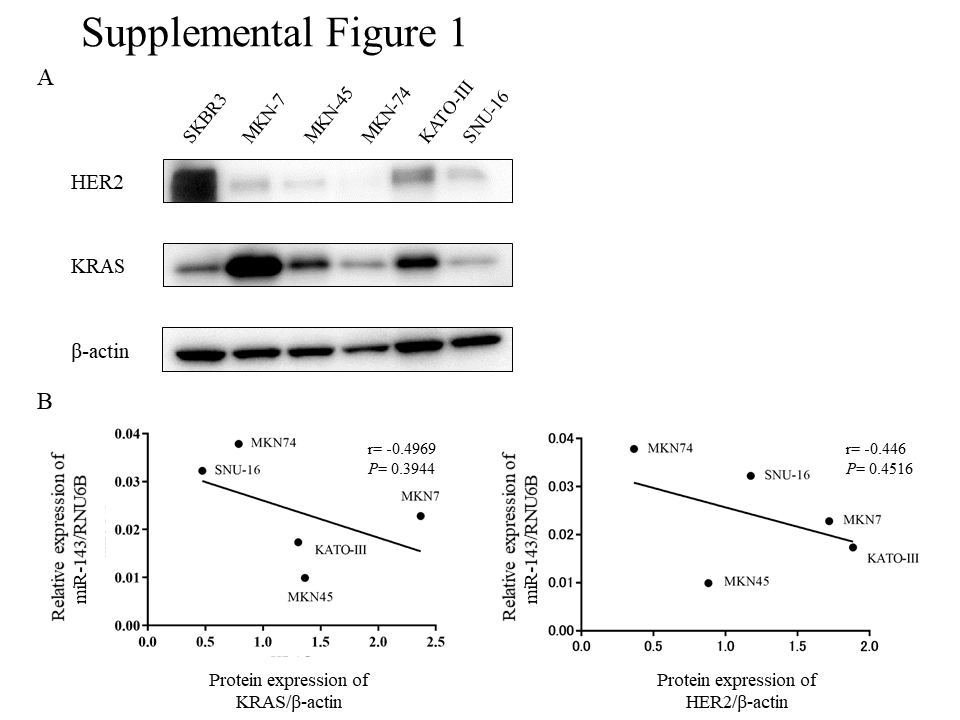

Supplement: Supplementary file 1 [file ijms-20-01697-s001.zip › supplementary figures/Supplementary Figure S1.tif]

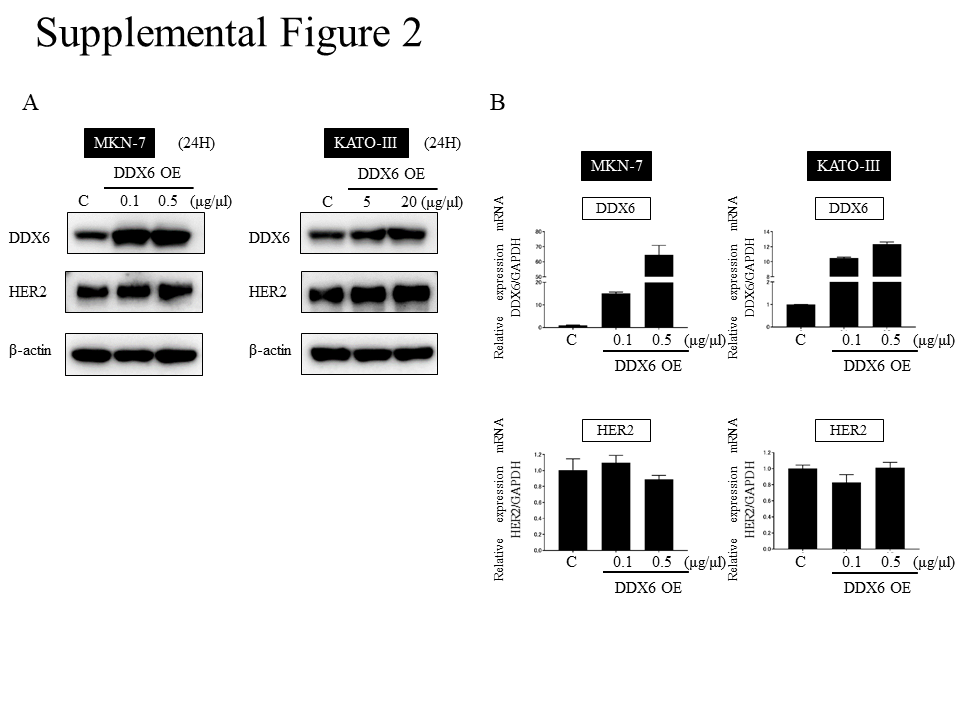

Supplement: Supplementary file 1 [file ijms-20-01697-s001.zip › supplementary figures/Supplementary Figure S2.tif]

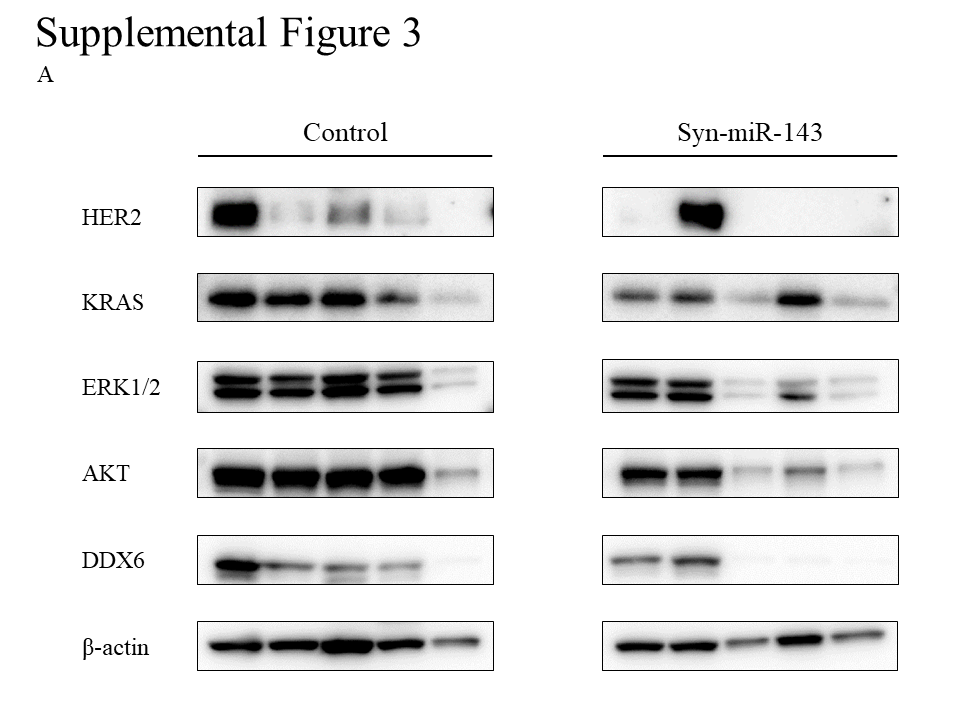

Supplement: Supplementary file 1 [file ijms-20-01697-s001.zip › supplementary figures/Supplementary Figure S3.tif]

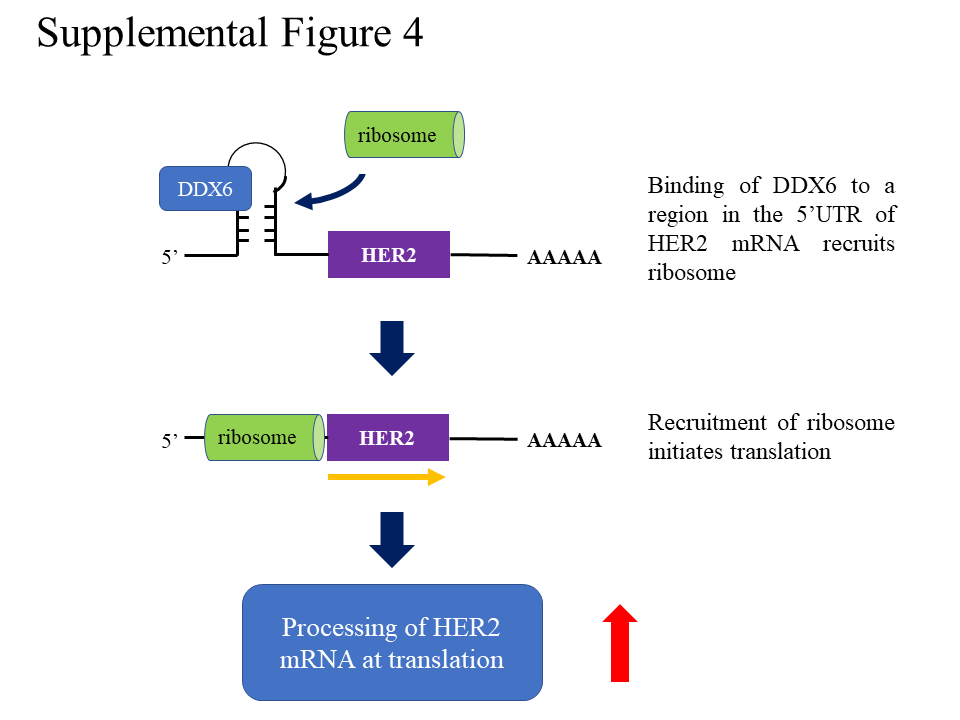

Supplement: Supplementary file 1 [file ijms-20-01697-s001.zip › supplementary figures/Supplementary Figure S4.tif]

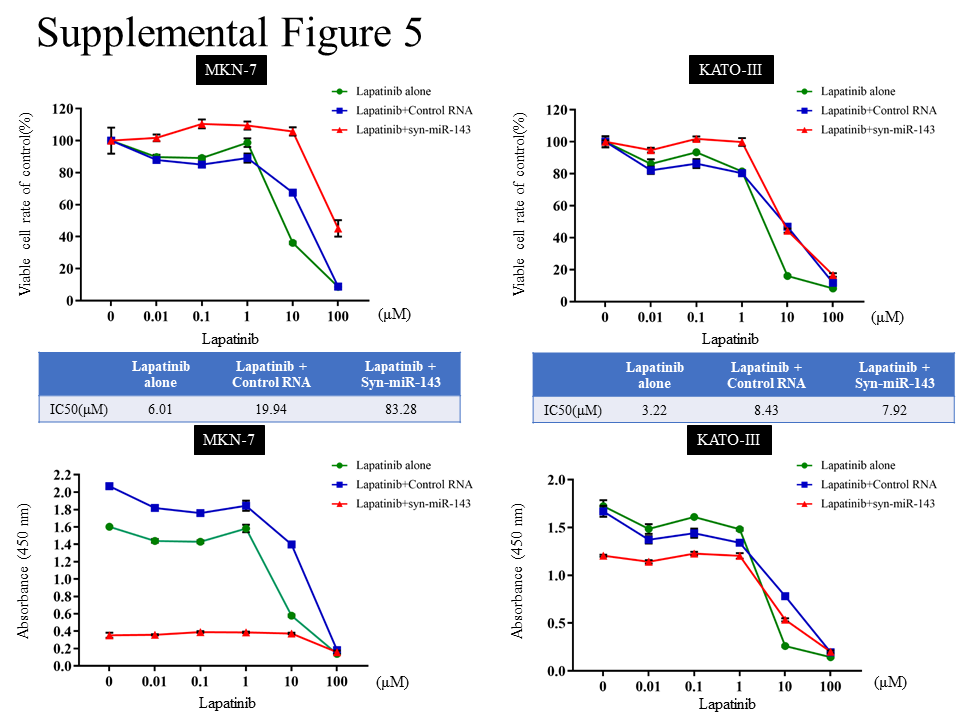

Supplement: Supplementary file 1 [file ijms-20-01697-s001.zip › supplementary figures/Supplementary Figure S5.tif]
